# Correlates of In-Hospital COVID-19 Deaths: A Competing Risks Survival Time Analysis of Retrospective Mortality Data

**DOI:** 10.1017/dmp.2021.85

**Published:** 2021-03-25

**Authors:** Ashish Goel, Alpana Raizada, Ananya Agrawal, Kamakshi Bansal, Saurabh Uniyal, Pratima Prasad, Anil Yadav, Asha Tyagi, RS Rautela

**Affiliations:** 1 Department of Medicine, UCMS and GTB Hospital, Delhi; 2 Hamdard Institute of Medical Sciences and Research, New Delhi, India; 3 UCMS and GTB Hospital, Delhi, India; 4 Department of Pedodontics and Preventive Dentistry, UCMS and GTB Hospital, Delhi, India; 5 Department of Anesthesia, UCMS and GTB Hospital, Delhi, India

**Keywords:** mortality, death, COVID-19, competing risks, recovery

## Abstract

**Introduction:**

Several aspects of the coronavirus disease 2019 (COVID-19) pandemic remain ambiguous, including its transmission, severity, geographic, and racial differences in mortality. These variations merit elaboration of local patterns to inform wider national policies.

**Methods:**

In a retrospective analysis, data of patients treated at a dedicated COVID hospital with moderate and severe illness during 8 wk of the pandemic were reviewed with attention to mortality in a competing risks framework.

**Results:**

A total of 1147 patients were hospitalized, and 312 (27.2%) died in hospital. Those who died were older (56.5 vs 47.6 y; *P* < 0.0001). Of these, 885 (77.2%) had tested positive on reverse transcriptase polymerase chain reaction (RT-PCR), with 219 (24.2%) deaths (incidence rate, 1.9 per 100 person-days). Median time from onset of symptoms to death was 11 days. A competing risks analysis for *in-hospital death* revealed an adjusted cause-specific hazard ratio of 1.4 for each decade increase in age.

**Conclusions:**

This retrospective analysis provides broad patterns of disease presentation and mortality. Even COVID test-negative patients will receive treatment at dedicated facilities, and 33% presenting cases may die within the first 72 h, most with comorbid illness. This should be considered while planning distribution of services for effective health-care delivery

The severe acute respiratory syndrome coronavirus (SARS CoV-2) has impacted every aspect of human life globally in a few months after the World Health Organization (WHO) declared it a public health emergency of international concern. As the world prepares itself to receive the vaccines developed at an unprecedented speed and other novel therapies, several questions remain unanswered, even while newer ones emerge every day.

While people struggled with a psychological disaster in addition to a physical illness, the definition of what constituted *a COVID death* remained obscure, with considerable regional variations, for a long time.^1–3^ However, the variations in mortality rates and disease severity with geographic and racial diversity cannot be attributed to procedural differences alone. During the past year, there have been more than 90 million global cases of COVID-19, and nearly 2 million deaths.^4^

While the case fatality rate (CFR) has been high in countries such as Yemen (29%), Mexico (8.7%), Hubei, China (6.6%), and United Kingdom (2.6%), it has been markedly lower in New Zealand (1.1%), Turkey (1%), and other neighboring countries of India such as Nepal (0.7%) and Sri Lanka (0.49%).^5^ India has had 150,000 deaths in 10 million cases, which is far lower than in many countries, and a consistent CFR under 2%.^5^ In addition to a remarkable variation in the CFR between countries, considerable differences for in-hospital mortality have been noted, ranging from 20% to 50% in different series.^6^ While interpretation of the CFR itself is complex, it has been proposed that CFR computation and interpretation should include estimation of the time interval between the onset of illness and death.^7^ A reliable estimate of CFR maybe possible only after right censoring for the duration. Additionally, limited testing and challenges in the attribution of the cause of death could be confounders in estimating the true CFR from the disease.

The variations in mortality have been attributed to racial differences, including immunity, demographic characteristics involving age composition of the affected population, hospitalization criteria, availability of intensive care, administrative decision-making including early and effective social lockdown, effectiveness of health care, testing strategies used, as well as the definition used to identify *a COVID-death*. The day-to-day variation in Wuhan had even been associated to changes in temperature and humidity.^8^ Not only does the quality of reporting influence the recording of deaths, but the phase of the pandemic could also determine its intensity. While the impact of early implementation of public health measures cannot be over-emphasized, it is noteworthy that the role of diet, meal timing, glutathione deficiency, ethnicity, genetic susceptibility, blood group, chest diameter, and air pollution have all been invoked in the literature.^9–19^ Yet, none of these are entirely convincing, and the comprehensive picture continues to elude us. Reports from China, Italy, and the United States have suggested that risk factors for severe disease include older age and presence of comorbid conditions such as diabetes and hypertension.^20–24^

The assumption that older patients in India would have a similar risk of severe disease and unfavorable outcome has guided the Indian public health response. However, initial Indian data suggested that younger people died at a higher rate than was expected (37%) from the Italian studies.^25,26^ Health-related data during COVID times are sparse, despite marked regional differences in public health statistics related to cases, recovery, and death. The reported positivity rate in cities such as Delhi, Mumbai, and Ahmedabad has been nearly 5-times higher than the national average.^25^ This diversity underscores the need to closely analyze local data to determine unique characteristics that could guide national policy and local action.

The present study aimed to analyze the demographic and clinical profile of patients with COVID-19 who succumbed to illness in our hospital while accounting for the effect of gender, clinical presentation, and comorbid conditions on mortality.

## Methods

A retrospective analysis of patient data for individuals hospitalized during 8 wk of the pandemic between June 20 and August 15, 2020, was conducted at our large tertiary-care public facility in Delhi, operating as a COVID-19–dedicated hospital during this period. Data collected for national/state level monitoring were analyzed after an approval by the Institutional Ethics Committee for human research.

The exposure variables for analysis included clinical features such as cough, breathlessness, fever, myalgia, conjunctivitis, headache, etc. Presence of pre-existing comorbid conditions such as diabetes, hypertension, chronic obstructive airway disease (COAD), chronic kidney disease (CKD), cardiovascular disease (CVD), etc. was recorded. The dates of onset of symptoms, hospitalization, deterioration in health condition, discharge, and death were noted.

The data were examined using Stata (version 13, Stata Inc, USA) and were interrogated to determine association of age, sex, clinical presentation, and presence of comorbidity with outcome. Age was analyzed as a continuous variable and was also recoded into categories defined by decades where relevant. Groups were determined by outcome (discharge or death) and by COVID-19 test report (positive or negative). Differences across groups were examined using chi-squared test or *t*-test as applicable. A survival time analysis using the duration of illness from symptom onset until outcome was carried out and Kaplan-Meier survival estimates were computed. A competing risks survival time analysis was conducted for *in-hospital death* and discharge, and sub-distribution hazard ratios are reported.

## Results

**Figure 1.**
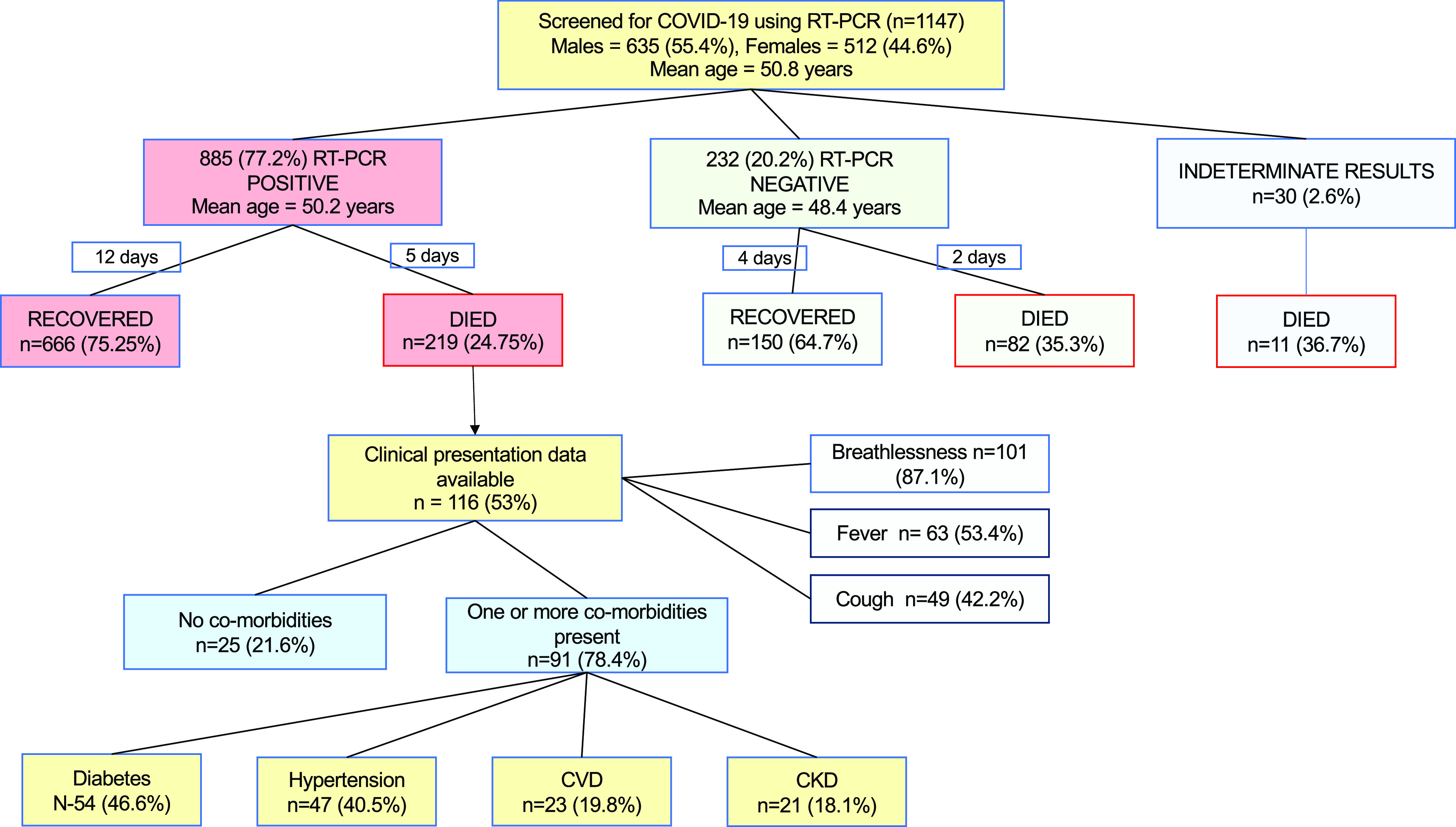
Flow diagram of the study.

A total of 1147 adult patients with a mean age of 50.0 y (±16.5 y) were hospitalized to our facility between June and August with symptoms suggestive of COVID-19 illness. Of these, 635 (55.4%) were males. A flow diagram of the study and the results is presented in Figure 1.

As many as 885 (77.2%) patients were confirmed positive on reverse transcriptase polymerase chain reaction (RT-PCR) and were not significantly older (50.2 ± 15.9 y) than the 232 who tested negative (48.4 ± 18 y; *P* = 0.15). Results of 30 (2.6%) tests were not available.

A total of 312 (27.2%) deaths were reported during the observation period in our hospital. Proportion of death among 885 who tested COVID-19 positive (*n* = 219; 24.8%) was lower than among the 232 who tested negative (*n* = 82; 35.3%; *P* = 0.001). Among those who tested positive, males had a higher probability of death (*n* = 138; 28.7%) compared to females (*n* = 81; 20.1%, *P* = 0.004). Among the COVID-19 positive patients, it was noted that more than a third of the patients (*n* = 81; 37%) died within the first 72 h of hospitalization.

**Figure 2.**
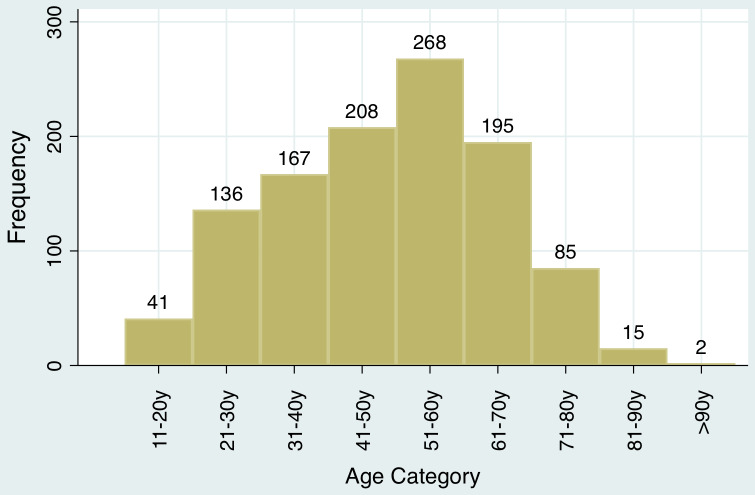
Age distribution of COVID-19 patients.

After adjusting for sex and COVID-19 test status, advancing age was found to be significantly associated with death. Each decade increase in age was associated with an increase in odds of 1.4 times (*P* < 0.001; 95% confidence interval [CI], 1.3-1.6). The age distribution of COVID-19 patients is presented in Figure 2.

**Figure 3.**
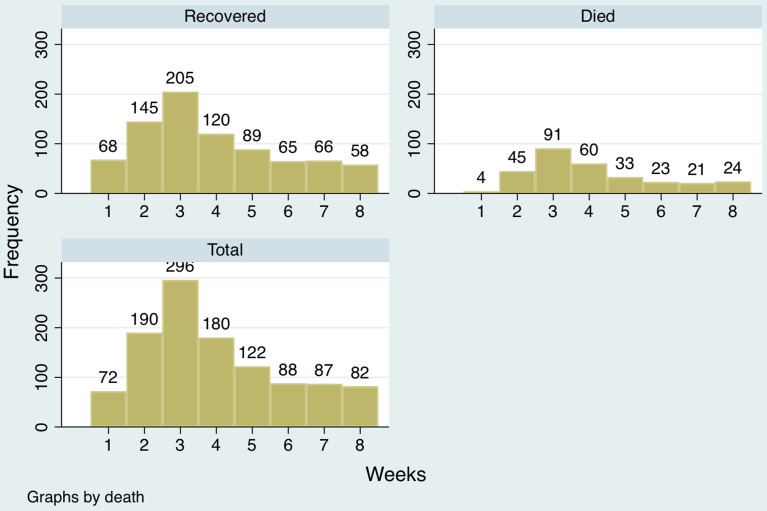
Distribution of hospital admissions.

Among the COVID-19 patients who tested positive, the 219 patients who died were significantly older (58.8 ± 13.7 y) than the other 666 who recovered (47.3 ± 15.6 y; *P* < 0.001). The mean age at the time of death did not differ by sex. A distribution of hospital admissions across the observation period is presented in Figure 3.

**Figure 4.**
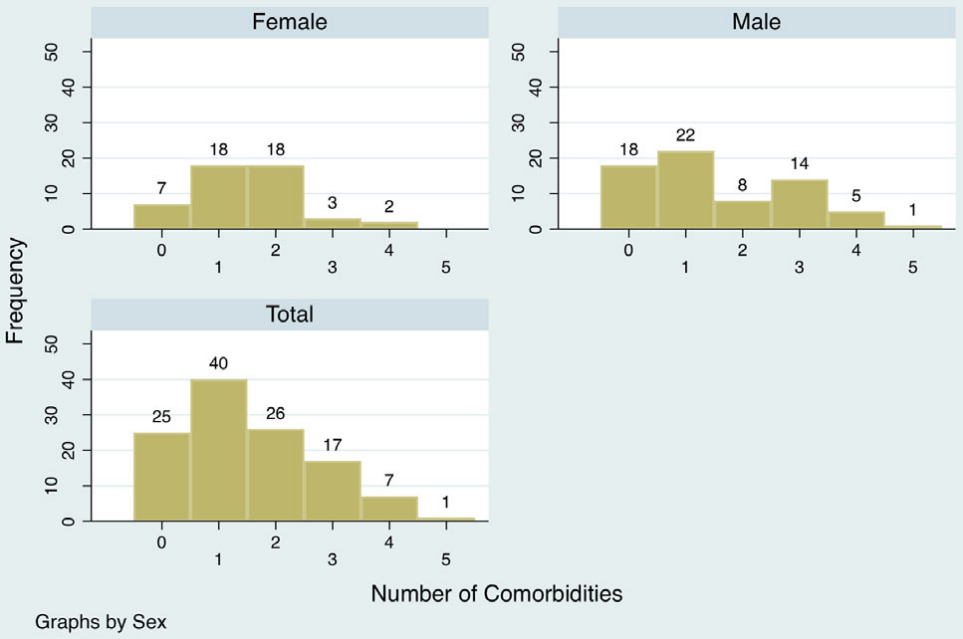
Comorbidity in COVID-19 patients who died.

Data on clinical presentation were available for 116 patients who had died. Most (*n* = 101; 87.1%) had presented to the hospital with breathlessness, while fewer had fever (*n* = 63; 54.3%) and cough (*n* = 49; 42.2%). Of these, 61 patients (53.5%) fulfilled the definition of severe acute respiratory illness (SARI) at the time of hospitalization. The pre-existing comorbid conditions included diabetes (*n* = 54; 46.6%), hypertension (*n* = 47; 40.5%), CKD (*n* = 21; 18.1%), and CVD (*n* = 23; 19.8%). Seven (6%) patients had a past history of tuberculosis, and 2 (1.7%) had COAD. Ninety-one (78.4%) patients had at least 1 or more comorbid illness. A distribution of comorbid conditions among the COVID-19 patients who died is presented in Figure 4.

**Figure 5.**
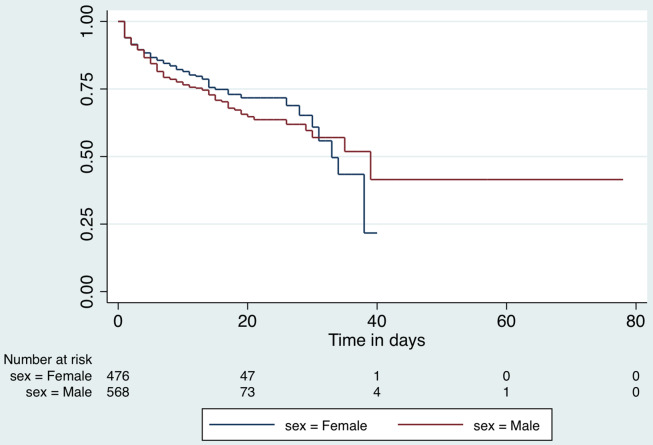
Kaplan-Meier survival function for hospital stay by sex.

**Figure 6.**
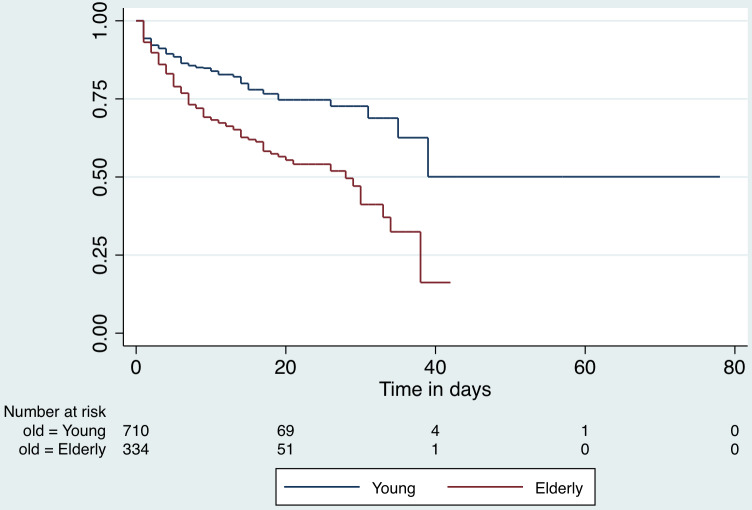
Kaplan-Meier survival function for hospital stay by age category.

Patients contributed 11,287 observation days for a survival-time analysis for the duration of hospital stay. The median hospital stay for all patients included in the analysis, was 8 days (interquartile range [IQR], 3-14 d). Those who died in the hospital had a mean stay of 6 d (95% CI, 5.1-6.7), while those who recovered remained hospitalized for 12 d (95% CI, 11.4-12.5; *P* < 0.001). The incidence rate of in-hospital death among the COVID-19 positive patients in hospital was 1.9 per 100 person-days. The Kaplan-Meier survival curves by sex are presented in Figure 5 and by age category (old ≥ 60 y; young < 60 y) in Figure 6.

**Figure 7.**
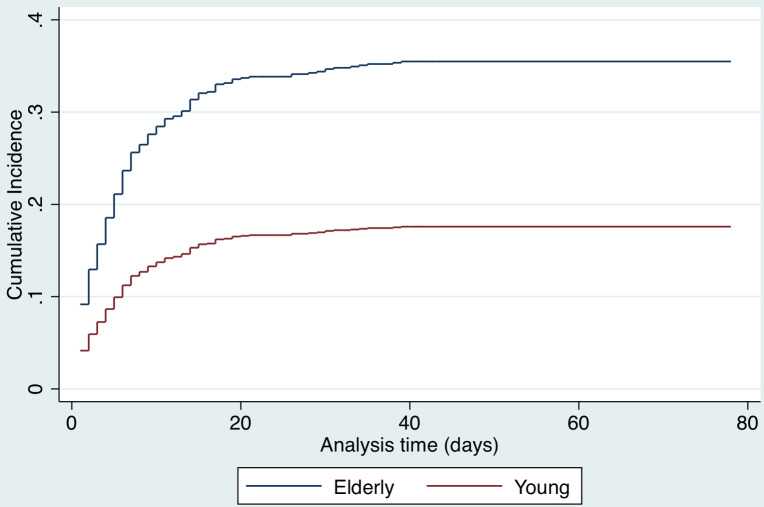
Competing risks analysis for in-hospital mortality.

**Figure 8.**
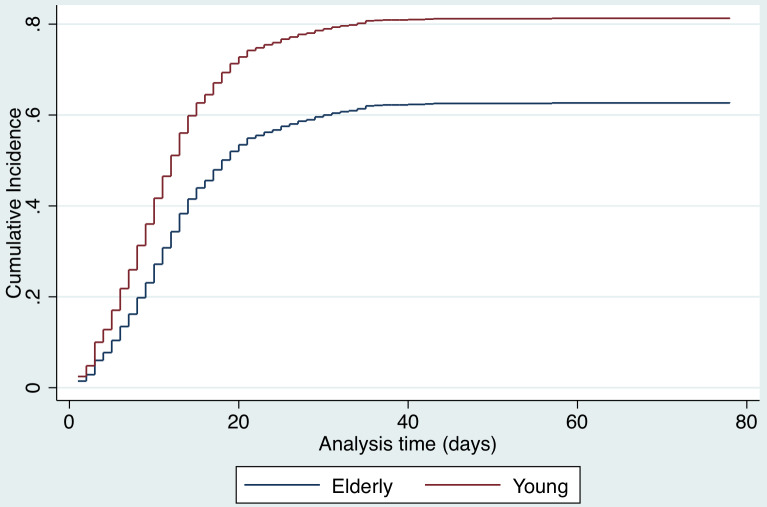
Competing risks regression analysis for discharge.

A competing risks analysis for *in-hospital death* reveals a cause-specific hazard ratio of 1.4 (95% CI, 1.3-1.5) for each decade increase in age after adjusting for sex and COVID-19 test result. The cumulative incidence function distribution by age category (old vs young) for in-hospital death is presented in Figure 7. A competing risk analysis for *discharge* reveals a cause-specific hazard ratio of 0.8 (95%CI, 0.76-0.84) for each decade increase in age after adjusting for sex and COVID-19 test result. The cumulative incidence function distribution by age (old vs young) for discharge is presented in Figure 8.

**Figure 9.**
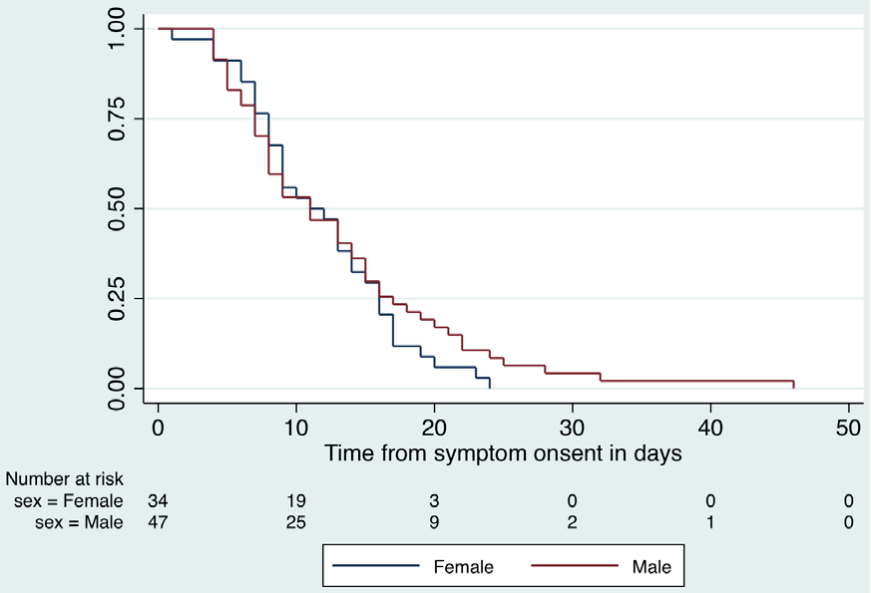
Kaplan-Meier survival function for death resented by sex.

A total of 81 COVID-19 positive patients, who succumbed to illness, contributed 1016 observation days for survival analysis from onset of symptoms. Median time to death was 11 d (IQR, 7-16) from the onset of symptoms. The Kaplan Meier survival function for death from onset of symptoms is presented in Figure 9.

## Discussion

Understanding the factors associated with death has been an area of interest in recent pandemic times. Kobayashi et al. have argued that, in a disease such as COVID-19, a simple CFR may not be enough to understand the disease due to its peculiar nature that merits estimation of time to death in view of several cases continuing to be sick at any given time.^7^

By this time, there are previous data from developed countries describing associations of mortality in COVID-19. A large body of data regarding the profile of patients appears from Wuhan,^8,24,27–35^ followed by a few European countries^6,21,22,36–40^ and scantily from other developing countries.^41–44^

We have observed a CFR of 24.8%, which is lower than 29.4% reported in the only available mortality related analysis from India by Tambe et al.^41^ However, our results reflect the distribution from a 5-times larger cohort than the earlier study (1147 vs 197). The mortality noted by us is close to that reported by Karagiannidis et al. in a large cohort analysis across German hospitals including 10,021 hospitalized patients.^6^ A much lower mortality of 16.1% among 701 patients was reported by Cheng et al. from their hospital in Wuhan.^45^

The adverse effect of advancing age on mortality has been identified in many countries.^29,39,40,46–48^ The presence of pre-existing comorbidity has been established as a risk for severe disease and mortality.^29,37,39,40,43,44,46–50^ Our findings are in sync with the international literature with hypertension and diabetes emerging as the commonest comorbid illnesses. In a slight deviation from these consistent results, the Italian model to predict death noted that while co-existing diabetes, CKD, and COPD exaggerated the risk of death, hypertension did not.^46^ This may be explained by the variation in severity and control of hypertension. Also, Rottoli et al. identified obesity as another risk factor in a retrospective analysis of 482 Italian patients.^51^ Obesity could be a markedly different health variable in developing versus developed countries. Using a novel health analytics platform, Williamson et al. have reported that deprivation and presence of comorbid illness conferred greater risk of mortality.^47^

Although our study does not include laboratory parameters for their evaluation as prognostic markers there are previous data for the same.^29,36,37,40,52–59^ Increase in indicators such as neutrophils, neutrophil-leukocyte ratio, lactate dehydrogenase, troponin, d-dimer, C-reactive protein, creatinine, urea, interleukins, and markers of cardiac injury have been associated with a poorer prognosis.

We have noted an adjusted hazard ratio of 1.4 for mortality with every decade increase in age. The association of increasing age and gender variation in COVID-related mortality has been noticed by Zhang et al. in a retrospective review of 82 deaths in Wuhan, with 65.9% of the victims being men and 80.5% older than 60 y.^48^ In similar experiences from Milan and Spain, older persons had a greater chance of dying.^39,40^

We note that hospital stay was longer for survivors compared to non-survivors, irrespective of positivity on RT-PCR test for COVID-19. Karagiannidis et al. have also reported that a hospital stay of more than 18 days was associated with increased chances of survival.^6^ This could be an encouraging finding when clinically attending to patients with prolonged hospital stay.

In an earlier analysis from our center, we have reported that 7.6% patients presenting with mild symptoms in the early pandemic phase in our city were found positive for COVID-19.^42^ The positivity rate among the moderate-severely ill patients who needed hospitalization in the present phase of the pandemic appears much higher at 77%. Also, the patients presenting during the early phase for testing were a decade younger (41 y) than those who needed hospitalization currently (50 y). This serves to highlight the changing nature of health services required as the pandemic continues to evolve, and is vital to planning services in any country.

The present study is the first study in which mortality from COVID-19 illness has been evaluated in a competing risk analysis framework and is also the largest series of patients who succumbed to the COVID-19 illness from India. Our study establishes that COVID-19–positive patients did not have a higher probability of death than those who were negative but presented with similar symptoms. In addition, we report that patients who are COVID-19 positive have a longer hospital stay after adjusting for age, sex, and outcome. We recognize the limitations in our study posed by gaps in our health-related data or indeterminate test reports and suggest that electronic medical records and hospital information systems could make data collection in developing countries more robust. It is interesting to note that, despite remarkable differences in the CFR in our country, the age and comorbidity profile of our patients is strikingly similar to the Western literature. Studies focusing on other key parameters, including host immunity, virus characteristics, and service delivery, would be able to ascertain the causes of lower mortality rates being reported from our region.

## Conclusions

This retrospective analysis allows us to provide answers to some broad patterns of disease presentation and its progression among different groups of individuals (differentiated by age and sex). Nearly 1 in 4 patients even at a dedicated facility may be COVID-19 negative and often too sick to shift to alternate facilities. Clinicians and administrators need to make arrangements to segregate and manage these individuals. Nearly 1 in 3 patients who succumb to illness in the hospital are likely do so within the first 72 h. In addition, those who die are likely to be older, with each advancing decade, conferring additional significant mortality risk. This should be considered while planning distribution of services. Nearly 4 of 5 patients who succumb to illness have associated comorbidity. Our findings reiterate the need for early diagnosis, testing, and multidisciplinary management. Within clinical facilities these inferences shall allow informed decision making for seamless, effective, and sustained health-care services to reduce mortality.
